# Comparison of two technics of cardiopulmonary bypass (conventional and mini CPB) in the trans-and postoperative periods of cardiac surgery

**DOI:** 10.5935/1678-9741.20150046

**Published:** 2015

**Authors:** Sergio Nunes Pereira, Izabelle Balta Zumba, Micheline Sulzbacher Batista, Daniela Da Pieve, Elisandra dos Santos, Ralf Stuermer, Gerson Pereira de Oliveira, Roberta Senger

**Affiliations:** 1 Centro de Ciências da Saúde da Universidade Federal de Santa Maria (CCS – UFSM), Santa Maria, RS, Brazil.; 2 Universidade Federal de Santa Maria (UFSM), Santa Maria, RS, Brazil.; 3 Hospital Universitário de Santa Maria (HUSM), Santa Maria, RS, Brazil.

**Keywords:** Cardiopulmonary Bypass, Perfusion, Postoperative Period

## Abstract

**Objective:**

This study aimed to compare the effects of two different perfusion techniques: conventional cardiopulmonary bypass and miniature cardiopulmonary bypass in patients undergoing cardiac surgery at the University Hospital of Santa Maria - RS.

**Methods:**

We perform a retrospective, cross-sectional study, based on data collected from the patients operated between 2010 and 2013. We analyzed the records of 242 patients divided into two groups: Group I: 149 patients undergoing cardiopulmonary bypass and Group II - 93 patients undergoing the miniature cardiopulmonary bypass.

**Results:**

The clinical profile of patients in the preoperative period was similar in the cardiopulmonary bypass and miniature cardiopulmonary bypass groups without significant differences, except in age, which was greater in the miniature cardiopulmonary bypass group. The perioperative data were significant of blood collected for autotransfusion, which were higher in the group with miniature cardiopulmonary bypass than the cardiopulmonary bypass and in transfusion of packed red blood cells, which was higher in cardiopulmonary bypass than in miniature cardiopulmonary bypass. In the immediate, first and second postoperative period the values of hematocrit and hemoglobin were higher and significant in miniature cardiopulmonary bypass than in the cardiopulmonary bypass, although the bleeding in the first and second postoperative days was higher and significant in miniature cardiopulmonary bypass than in the cardiopulmonary bypass.

**Conclusion:**

The present results suggest that the miniature cardiopulmonary bypass was beneficial in reducing the red blood cell transfusion during surgery and showed slight but significant increase in hematocrit and hemoglobin in the postoperative period.

## Abstract (pt)

**Objetivo:**

Este estudo teve como objetivo comparar os efeitos de duas técnicas de perfusão diferentes: convencional (CEC) e mini (MCEC) em pacientes submetidos à cirurgia cardíaca no Hospital Universitário de Santa Maria - RS.

**Métodos:**

Foi realizado um estudo retrospectivo, transversal, baseado em dados coletados dos pacientes operados entre 2010 e 2013. Foram analisados ​​os registros de 242 pacientes divididos em dois grupos: Grupo I - 149 pacientes submetidos à circulação extracorpórea e Grupo II - 93 pacientes submetidos à minicirculação extracorpórea.

**Resultados:**

O perfil clínico dos pacientes no pré-operatório foi semelhante nos grupos circulação extracorpórea e minicirculação extracorpórea sem diferenças significativas, exceto na idade, maior no grupo minicirculação extracorpórea. Os dados perioperatórios foram significativos de sangue coletadas para autotransfusão, que foram maiores no grupo com minicirculação extracorpórea do que a circulação extracorpórea e na transfusão de concentrado de hemácias, superior em circulação extracorpórea do que em minicirculação extracorpórea. Nos valores pós-operatório imediato, primeiro e segundo, os níveis de hematócrito e da hemoglobina foram maiores e significativos na minicirculação extracorpórea que no circulação extracorpórea, embora o sangramento no primeiro e segundo dias pós-operatórios tenha sido maior e significativo no minicirculação extracorpórea que no circulação extracorpórea.

**Conclusão:**

Os resultados sugerem que a minicirculação extracorpórea foi benéfica em reduzir a transfusão de células vermelhas do sangue durante a cirurgia e houve discreta, mas significativa, elevação do hematócrito e hemoglobina no pós-operatório.

**Table:** 

**Abbreviations, acronyms & symbols**	
1^st^ POP	First postoperative day
2^nd^ POP	Second postoperative day
ACT	Activated coagulation time
CABG	Coronary artery bypass grafting
CPB	Cardiopulmonary bypass
IOPP	Immediate postoperative period
MCPB	Miniature cardiopulmonary bypass
SIRS	Systemic inflammatory response syndrome

## INTRODUCTION

Cardiac surgery had major limitations in the beginning, in the early twentieth century, for not being able to stop and open the heart to treat intracardiac lesions. However, from the development of an artificial heart-lung machine^[1]^, showed great progress, especially with the development of cardiopulmonary bypass, progressively improved until 1954, when its use in humans has started^[2]^. Since that time this technique has become widespread worldwide and known as the largest contribution to cardiac surgery and cardiology for the world^[3-5]^. In Brazil this technique started in 1955^[6-7]^, followed by several surgeons^[8-12]^, which put the country in an international leading position in cardiovascular surgery with important contributions to the development and improvement of perfusion. However, with the method came the challenges to circulate blood into metal and plastic surfaces. The contact of blood with this surfaces predisposes to changes in blood components, such as red cells, white cells, platelets, and plasma lipoproteins, that can suffer degradation and partial destruction of these elements, resulting in anemia and tissue inflammatory reactions as the systemic inflammatory response syndrome (SIRS)^[13]^, need for transfusion with homologous red blood cells^[14]^ and increased risk of postoperative infection^[15]^. Because of this situation, various techniques were used, such as coronary artery bypass grafting without cardiopulmonary bypass (CPB) in 1955^[16]^, and later with other surgeons^[17-20]^, with good results in relation to the CPB. But these technical limitations were reported as difficult as revascularization of the lower wall of the left ventricle, large cardiomegaly and severe heart failure. In these situations, the technique often resulted in incomplete revascularization^[21]^. Another option found to the problems of CPB was to minimize the volume of the infusion, with the miniaturization of the cardiopulmonary bypass (MCPB). Afterwards, several studies published comparing the CPB with off pump surgery and the MCPB, finding lower presence of hemodilution, coagulopathy^[21]^ need for transfusion of red blood cells^[22-27]^ and lower systemic inflammatory reaction in the surgery without CPB and MCPB in relation to the CPB^[28]^. It was also observed higher hemoglobin levels in MCPB than in CPB^[26]^. When comparing off-pump surgery and MCPB, the following effects were described: similar level of inflammatory response^[25]^, but more controlled surgical field^[21]^, better coronary artery bypass grafting (CABG)^[25]^ and higher level of hemoglobin in the MCPB than in off-pump. Other authors considered not significant the difference between CPB and the MCPB for bleeding, renal injury, length of stay^[29]^ and evolution of low-risk patients^[30]^. In our Service, at the University Hospital of Santa Maria, from 2010, we began using MCPB in CABG surgery. In 2011, we added to this technique an autotransfusion equipment with hemoconcentration. This year, a resident of anesthesia and Master's student, comparing patients undergoing coronary artery bypass surgery found less need and lower volume of packed red blood cells in the autotransfusion group than in those without its use^[28]^. Based on this initial experience, we decided to perform a retrospective analysis with a review of medical records of patients undergoing consecutive cardiac surgeries in 2010 to 2013 period, regarding the effects of two types of cardiopulmonary bypass: (with conventional CPB and MCPB) on the results of clinical and laboratory parameters of the periods before, during and after surgery. Considering the fact that this study was retrospective, we analyzed mainly the clinical aspects and changes in the hemoglobin, hematocrit, platelets, complications related to bleeding and the need of red blood pack transfusion during surgery and in the postoperative period.

## METHODS

### Ethical considerations

This study was reviewed and approved by the Research Ethics Committee of the Federal University of Santa Maria, RS, with number CAAE: 21598213.1.0000.5346 and order number: 434.030. Date: 08/10/2013.

Data were collected in chips (Chart 1), whose items refer to clinical, surgical and laboratory parameters of the pre-, intra- and postoperative surgery, with emphasis on hematological aspects, bleeding and transfusions, regarding the patients underwent conventional CPB and MCPB. The preoperative data refer to clinical and laboratory parameters collected before surgery; the perioperative are related to the period from the beginning to the end of surgery. The postoperative period was subdivided into three sub-periods: the early postoperative period (POI) between arrival at the Intensive Cardiology Unit (ICU) and 6:00 pm the following day, and then the first postoperative day (24 h after) and the second PO (48 h after).

**Chart I:** Cardiac surgery sheet data of pre-, trans- and postoperative periods.

Hospital Records	Name	Age	Sex	Weight	BMI	Body Surface	Smoking	DM_2
SAH	COPD	Dislipidemia	Renal Insuff.	Previous AMI	Other	Cir. Date	Type of Surgery	Type ECC
LITA	B. S.	C.T.	Time ECC	End Ht	Pre Plat.	Initial ACT	Top Bleeding	Autol. Transf.
PRBC_Top	B. B.	End ACT	End Ht	End Hb	IP Bleed.	IP Ht	IP Hb	IP CHAD.
IP Plat.	1^st^ and 2^nd^ POP Bleed.	1^st^ and 2^nd^ POP Ht	1^st^ and 2^nd^ POP Hb	1^st^ and 2^nd^ POP Plat.	Hosp. Dis.		Obs: Complication	

BMI=body mass index; BS=body surface; DM 2= diabetes mellitus type 2; SAH=Hypertension; COPD=chronic obstructive pulmonary disease; Dyslipid=dyslipidemia; Renal F=renal failure; AMI=acute myocardial infarction; EF=ejection fraction; LITA=left internal thoracic artery; CABG=coronary artery bypass surgery; Clamp T=clamp time; Init Ht=initial hematocrit; Init Hb= initial haemoglobin; Pre Plat=previous platelets; Init ACT=activated clotting time; IP Bleed=intraoperative bleeding; Autol Tr=autologous transfusion; CRBC=concentrate of red blood cells; Blood Bal=blood balance; ACT: activated clotting time; Final Ht=final Hematocrit; Final Hb=final hemoglobin; Final Plat=final platelets; IPO bleed.=Bleeding pf immediate postoperative bleeding; IPO Ht=immediate postoperative hematocrit; IPO Hb=immediate postoperative hemoglobin; IPO CRBC= immediately postoperative CRBC; IPO Plat=Immediate postoperative period platelets; Bleed 1^st^ and 2^nd^ PO=Bleeding on the 1^st^ and 2^nd^ postoperative day; Ht 1^st^ and 2^nd^ PO=hematocrit on the 1^st^ and 2^nd^ postoperative day; Hb 1^st^ and 2^nd^ PO=hemoglobin on the 1^st^ and 2^nd^ postoperative day; Plat 1^st^ and 2^nd^ PO=Platelets on the 1^st^ and 2^nd^ postoperative day

In this study we analyzed the medical records of 242 patients who underwent surgery between 2010-2013, divided into two groups:

Group I (GI) - 149 patients undergoing surgery with conventional cardiopulmonary bypass (CPB).

Group II (GII) - 93 patients who underwent surgery with miniaturized cardiopulmonary bypass (MCPB).

**Fig. 1:**
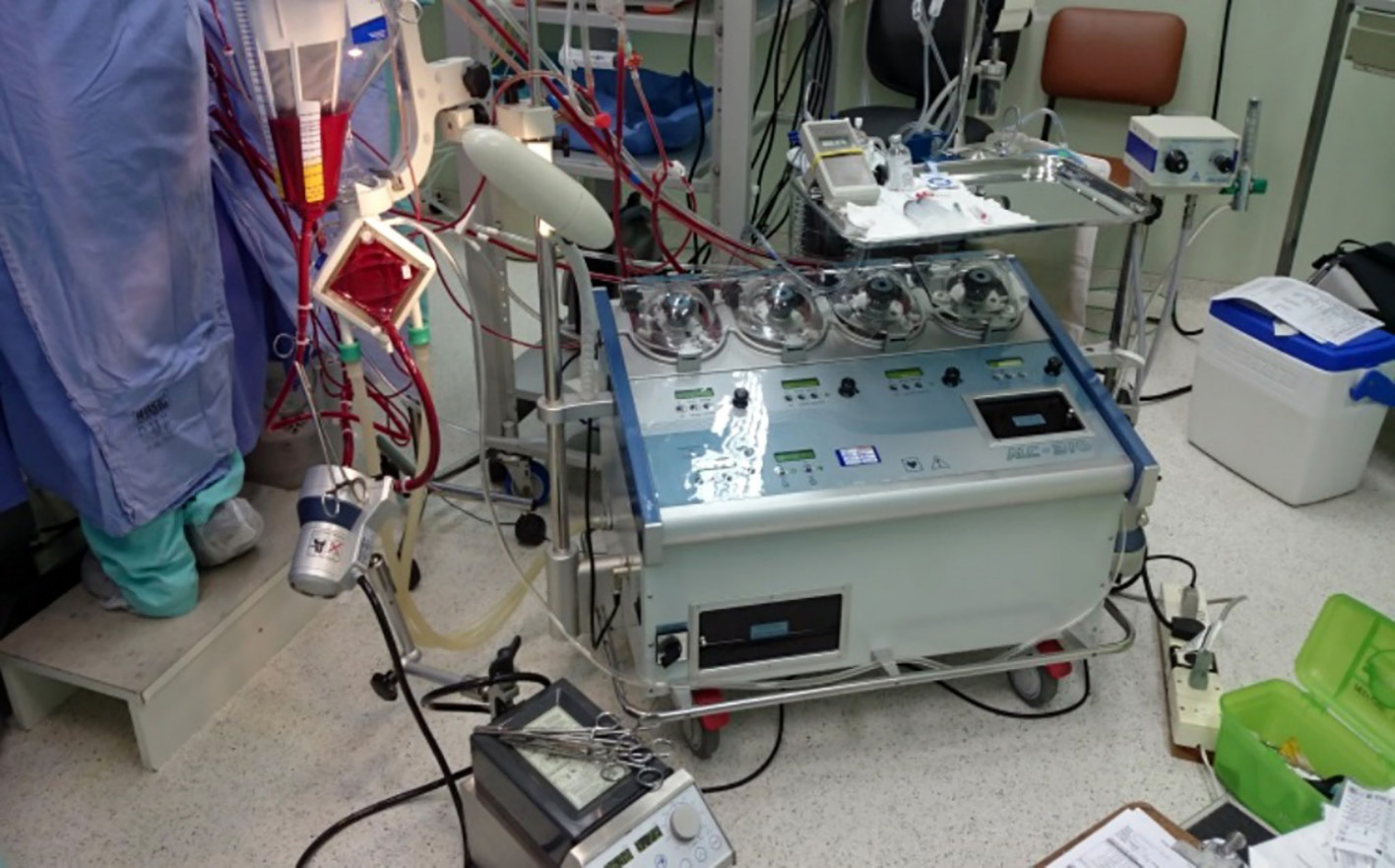
Maquet set consisting of mini bypass circuit connected to the centrifugal pump and the extracorporeal circulation machine.

**Fig. 2:**
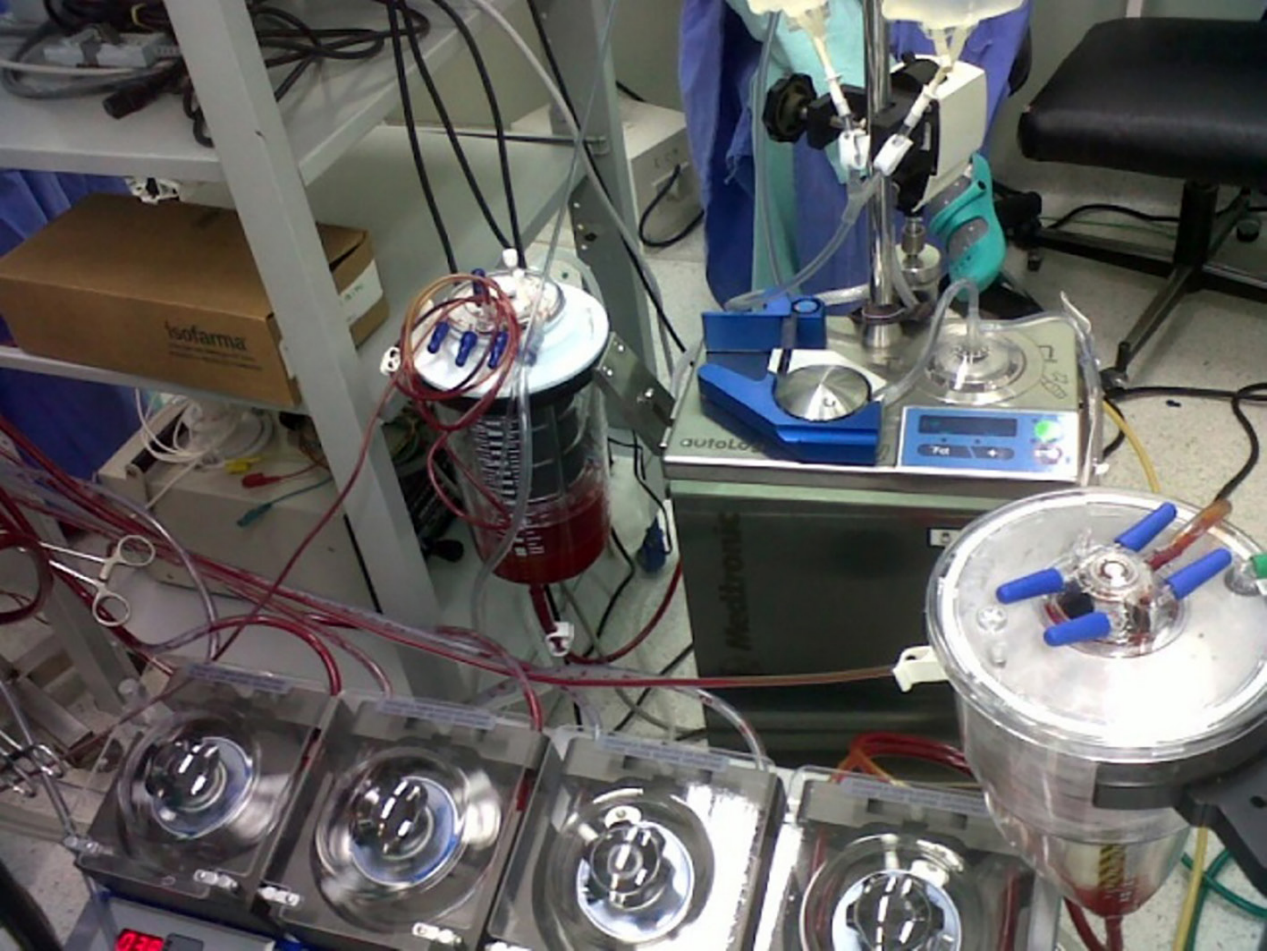
Bypass set with centrifugal pump coupled with the module of auto transfusion (Autolog).

The patients in GI underwent surgery with machine and conventional CPB circuits (Braile Biomédica^®^) and centrifugal pump (Maquet^®^). The GII, were operated circuit, centrifugal pump and MCPB Maquet^®^ machine (Figure 1). Auto transfusion was performed by using a device named Autolog (Medtronic^®^) (Figure 2), when deemed necessary by the surgeon. The surgical procedures were performed by the usual techniques, corresponding to each system. Autolog® was used in both types of perfusion: 148 in the CPB group and 88 in the MCPB group. Data were tabulated in spreadsheet (Excel 2010 Windows^®^), and analyzed using the statistical package (SPSS 15.0)^®^, with test application T Student for parameters with normal distribution and Mann Whitney test for abnormal distribution, considering the significance of *P*<0.05. Inclusion criteria were cardiac surgeries performed sequentially in the period 2010-2013, and the exclusion were emergency surgery, reoperation and complex surgeries such as aneurysms and aortic dissection, given that our initial aim was to compare these perfusion techniques in routine surgery.

## RESULTS

**Fig. 3:**
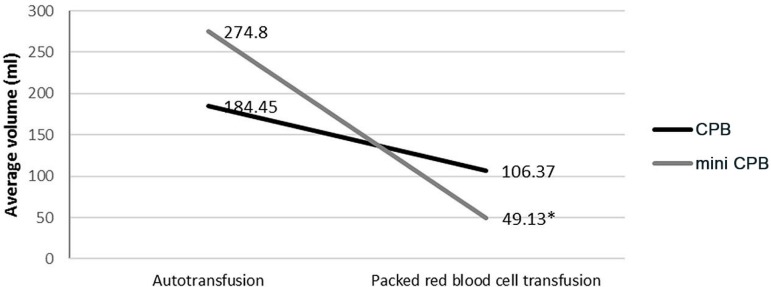
Comparison of the type of infusion and compared to autologous transfusion concentrated red blood cell.

The results were tabulated according to the analysis period: preoperative (Table 1), intraoperative (Table 2) and postoperative (Table 3). The clinical profile of patients preoperatively (Table 1) was similar in groups I (CPB) and II (mini CPB), differing only in age, the greater the mini CPB in relation to the CPB. (*P*<0.05). During the surgery (Table 2) there was no significant difference in duration of CPB and laboratory parameters and bleeding. There were significant differences in the collection of red blood cells for autologous transfusion (*P*<0.05) in the mini CPB in relation to the CPB and the volume of transfused red blood cells concentrate (Figure 3) was greater and significant in the CPB regarding the mini pump group (*P*<0.04).

**Table 1:** Preoperative clinical and anthropometric parameters in CPB and MCPB groups.

Nominal variables	G I (CPB) N (%)	G II (MCPB) N (%)	*P* Value
Gender			
Female	45 (30.2%)	27 (29.0%)	0.85
Male	104 (69.8%)	66 (71.0%)	
Smoking			
Ex	66 (44.6%)	45 (48.9%)	0.43
No	52 (35.1%)	25 (27.2%)	
Yes	30 (20.3%)	22 (23.9%)	
DM			
No	96 (64.9%)	55 (59.1%)	0.37
Yes	52 (35.1%)	38 (40.9%)	
SAH			
No	21 (14.1%)	14 (15.1%)	0.84
Yes	128 (85.9%)	79 (84.9%)	
COPD			
No	131 (87.9%)	83 (89.2%)	0.75
Yes	18 (12.1%)	10 (10.8%)	
Dyslipidemia			
No	43 (28.9%)	22 (23.9%)	0.40
Yes	106 (71.1%)	70 (76.1%)	
Renal Failure			
No	139 (93.3%)	84 (90.3%)	0.40
Yes	10 (6.7%)	9 (9.7%)	
Previous AMI			
No	84 (57.1%)	46 (49.5%)	0.25
Yes	63 (42.9%) CPB	47 (50.5%) MCPB	
Numeric variables	MEAN (± SD)	MEAN (± SD)	*P*-value
AGE	59.21 (11.63)	62.51 (9.55)	0.04*
BMI	26.73 (3.91)	26.91 (4.81)	0.80
Body Surface	1.83 (0.24)	1.85 (0.18)	0.79
EF	62.11 (10.71)	59.37 (13.27)	0.20

* P≤0.05. CPB=cardiopulmonary bypass; MCPB=mini cardiopulmonary bypass; DM=diabetes mellitus; SAH=Hypertension; COPD=chronic obstructive pulmonary disease; AMI=acute myocardial infarction; BMI=body mass index; EF=ejection fraction; SD=standard deviation

**Table 2:** Parameters of perioperative period.

Parameters	GI(CPB) Mean (± SD)	G II (MCPB) Mean (± SD)	%	*P* value
CPB TIME	149	93	-4.37	0.29
	94.06 (±25.85)	89.95 (±23.62)		
Initial Ht.	149	93	-0.75	0.69
	37.52 (±5.74)	37.24 (±5.10)		
Initial Hb	149	93	-1.62	0.41
	12.95 (±2.08)	12.74 (±1.83)		
Initial Platelets	146	93	-5.55	0.16
	219438.36 (±63383.341)	207249.76 (±58345.379)		
Initial ACT	148	92	0.05	0.63
	150.52 (±68.71)	150.59 (±85.21)		
Trans op bleeding	132	75	8.41	0.77
	556.39 (±449.39)	603.16 (±501.36)		
Autol transf	148	88	48.98	0.05*
	184.45 (±265.88)	274.80 (±345.97)		
PO Packed red blood cells	147	82	-53.81	0.04*
	106.37 (±211.969)	49.13 (±133.292)		
Final ACT	149	93	-0.75	0.21
	144.69 (±50.01)	143.60 (±63.32)		
Final Ht	149	93	5.03	0.06
	27.82 (±5.53)	29.22 (±5.87)		
Final Hb	149	93	2.57	0.12
	10.11 (±2.13)	10.37 (±1.91)		
Final Platelets	148	87	0.91	0.95
	167154.73 (±61929.863)	168678.16 (±60660.798)		

* P≤0.05. ACT=activated coagulation time; CPB=cardiopulmonary bypass; MCPB=mini cardiopulmonary bypass; SD=standard deviation; Hb=hemoglobin; Ht=hematocrit; Autol Tranf=autologous transfusion

**Table 3:** Parameters of postoperative period.

Parameters	GI(CPB) Mean (± SD)	G II (MCPB) Mean (± SD)	%	*P* Value
IPO Bleeding	149	90	-14.28	0.84
	325.10 (±312.52)	278.67 (±188.07)		
Ht IPO	146	92	4.93	0.03*
	32.14 (±5.28)	33.726 (±4.70)		
IPO Hb	145	92	4.57	0.03*
	10.73 (±1.71)	11.22 (±1.57)		
IPO Packed red blood cell	147	93	-32.92	0.30
	143.88 (±278.16)	96.51 (±198.94)		
IPO Platelets	142	91	-1.98	0.73
	162756.34 (±57825.240)	159538.46 (±54.421.260)		
1^st^ PO Bleeding	149	91	12.36	0.04*
	518.15 (±363.39)	582.20 (±343.93) *		
1^st^ PO Ht	149	93	5.09	0.04*
	30.43 (±5.0283)	31.979 (±4.2382) *		
1^st^ PO Hb	149	93	3.69	0.05*
	10.162 (±1.4594)	10.537 (±1.4544) *		
2^nd^ PO Bleeding	149	86	20.29	0.02*
	254.56 (±318.48)	306.22 (±289.22) *		
2^nd^ PO Ht	148	93	4.25	0.04*
	27.326 (±4.2496)	28.488 (±4.1622) *		
2^nd^ PO Hb	149	93	3.90	0.05*
	9.053 (±1.38)	9.406 (±1.39)		
1^st^ PO Platelets	149	93	6.10	0.83
	177234.90 (±172925.323)	188053.76 (±267024.765)		
2^nd^ PO Platelets	148	93	-11.74	0.67
	152662.16 (±143563.992)	134741.94 (±50825.127)		

* P≤=0.05. CPB=cardiopulmonary bypass; MCPB=mini cardiopulmonary bypass; Hb=hemoglobin; Ht=hematocrit; IPO=immediate postoperative; PO=perioperative; SD=standard deviation

*P<0.05; mini CPB=miniaturized cardiopulmonary bypass.

**Fig. 4:**
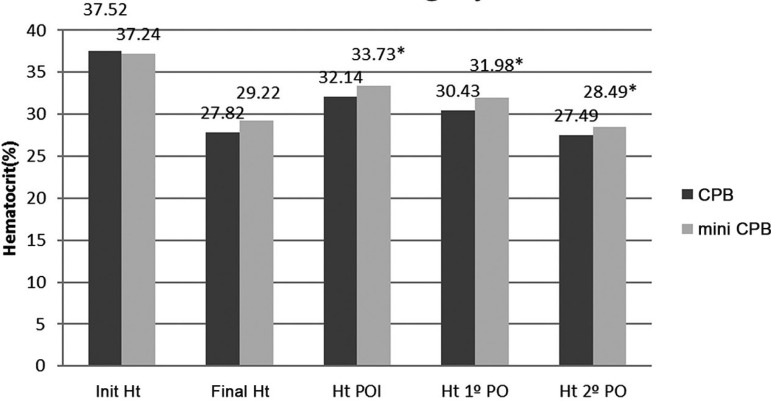
Hematocrit before, during, and after surgery.

**Fig. 5:**
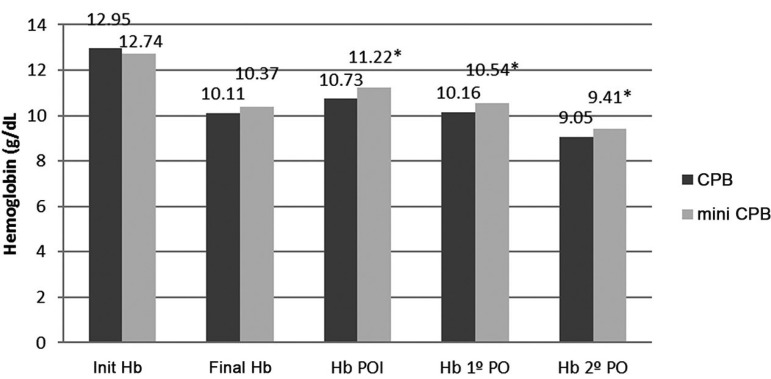
Hemoglobin before, during, and after surgery.

**Fig. 6:**
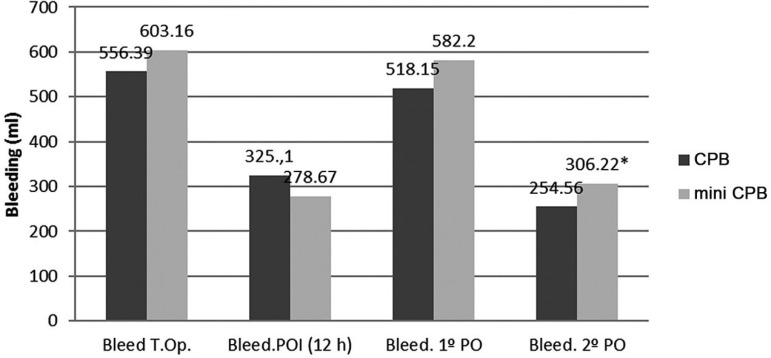
Bleeding trends in the trans-and postoperative periods.

**Fig. 7:**
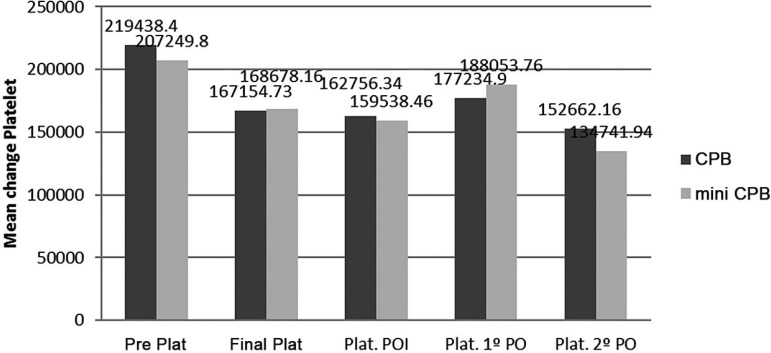
Evolution of platelets, during, and after surgery.

The immediate postoperative period (Table 3), with discrete higher values of hematocrit and hemoglobin in the mini CPB than in CPB (Figures 4 and 5), respectively, with significant differences (*P*<0.05). In the first and second postoperative period bleeding was also observed (Figure 6) and most significant in the mini CPB in relation to the CPB (*P*<0.05), however, despite this, the hematocrit and hemoglobin levels remained higher in mini CPB than in the CPB, with subtle differences, but significant (*P*<0.05). The complication about SIRS where referred in the Introduction, in the historic context of one important event that is related to CPB. However, in this study, we referred only the complications related to bleeding, need of RBC transfusion and change of erythrocytes, hematocrit, hemoglobin and platelets during surgery and postoperative period (Figure 7).

*P<0.05; Init Ht=initial hematocrit; Final Ht=final hematocrit; Ht POI=hematocrit on the immediate postoperative period; Ht 1 PO= hematocrit on the first day after surgery; Ht 2 PO= hematocrit on the second day after surgery.

*P<0.05; Init Heb=initial hemoglobin; Final Hb=final hemoglobin; Hb POI=hemoglobin on the immediate postoperative period; Heb 1 PO=hemoglobin on the first day after surgery; Heb 2 PO=hemoglobin on the second day after surgery.

*P<0.05; Bleed T.Op=postoperative bleeding; Bleed POI=bleeding on the immediate postoperative period; Bleed. 1º PO=bleeding on the first day after surgery; Bleed. 2º PO=bleeding on the second day after surgery.

*P>0.05; Pre Plat=previous platelets; Final Plat=final platelets; Plat POI=platelets on the postoperative period; Plat. 1º PO=platelets on the first day after surgery; Plat. 2º PO =platelets on the second day after surgery.

## DISCUSSION

Cardiac surgery had major limitations in the beginning, in the early twentieth century, for not being able to stop and open the heart to treat intracardiac lesions, but with the development of an artificial heart-lung machine and extracorporeal circulation, the technique has become known and disseminated worldwide as the largest contribution to cardiac surgery and for the world cardiology.

However, success was accompanied by problems such as hematological disorders^[13]^, cognitive, and systemic inflammatory reactions^[14]^ and infections^[15]^, resulting from direct blood contact with oxygen and non-endothelial surfaces like metal and plastic. These situations led to the search for solutions to work around the problem. Then emerged coronary revascularization without cardiopulmonary bypass^[16]^, technique followed by other surgeons, with good results in relation to the CPB^[14-16]^.

However, other challenges have emerged, especially in CABG as the difficult access to the lower wall of the heart, great cardiomegalies and severe heart failure, making it difficult to complete revascularization^[21]^. Another option was the miniaturization of cardiopulmonary bypass described as good alternative by several authors^[21-28]^ and restrictions by other researchers^[29-30]^.

Currently, the mini CPB is establishing itself as a suitable technique to reduce perfusion problems and the use of homologous blood. In our experience, the results for preoperative parameters (Table 1) showed no significant variation, with the exception of age, with a slight but significant increase in the group with mini CPB compared to conventional CPB (*P*<0.05). These findings are consistent with those of other authors^[25,26]^. In the perioperative period the volume of blood collected autotransfusion: 274.80 (±345.97) was 48.98% higher and significant (*P*<0.05) in the group with mini CPB in relation to the CPB: 184.45 (±265.88), but the average volume of transfused red blood cells was 106,37 ml (±211.97) in the pump group and 49.13 ml (±133.29) in the mini-pump group, with a difference of 57.24 ml (-53.81%) of this in relation to the CPB (*P*<0.04), showing a favorable effect on the mini CPB in relation to the CPB.

These findings suggest that patients of mini CPB, being autotransfused during surgery had less need for homologous blood transfusion than CPB. This result was similar to the data of other authors who found mean values of homologous blood units of 0.8 unit/patient in the groups with mini pump and off-pump, and 1.8 unit/patient in the CPB^[25]^. Another study showed that there was significant reduction in the volume of blood transfusion: 0.53±0.90 CH unit in mini CPB and 1.3±1.93 units in the CPB (*P*<0.05)^[26]^.

In our study the postoperative period was subdivided into three sub-periods: the early postoperative period (POI), first and second day after surgery, with the intention to better assess the effects of infusion at this stage after surgery. The results of these periods were: POI - Bleeding data, the use of concentrated red blood cells and platelets were not significant, but the hematocrit and hemoglobin had mild elevation, but significant (*P*<0.03). The first and second PO - bleeding was slightly more pronounced in the mini CPB in relation to the CPB (*P*<0.04), but despite this, hematocrit and hemoglobin remained higher in mini CPB in relation to the CPB (*P*<0.05). However, it is remarkable that this bleeding did not contribute to the reduction of hematocrit and hemoglobin.

These results are similar to those by other authors who also reported high hematocrit^[22]^ and hemoglobin^[26]^, after the use of mini CPB in relation to the CPB. In our research, the comparison between the two perfusion techniques showed better data on mini CPB in relation to the CPB, as referred to CPB and transfusion in the perioperative period and the results of red blood cells and hemoglobin, with statistically significant values​​. The results suggest that the mini CPB was beneficial in reducing the transfusion of packed red blood cells and higher levels of hematocrit and hemoglobin in trans- and postoperative period as mentioned in the literature. More studies are needed on the influences of the type of cardiopulmonary bypass and the use of autotransfusion and we are now working in in this matter to include in another study.

## CONCLUSION

In our study, the comparison between the two types of perfusion showed better data in the MCPB, from the first period in which the patient was referred to bleeding and transfusion in the perioperative period and the results of red blood cells and hemoglobin, with statistically significant values. The results suggest that the MCPB is beneficial for the reduction of perioperative bleeding, showing higher levels of hematocrit and hemoglobin in trans- and postoperative periods, and especially on reducing the use of concentrated homologous red blood cells, as reported in the literature. We are aware that this matter needs more studies.

**Table:** 

**Authors’ roles & responsibilities**	
SNP	Analysis and/or interpretation of data; final approval of the manuscript; study design; manuscript writing or critical re-view of its content
IBZ	Analysis and/or interpretation of data; implementation of projects and/or experiments
MSB	Analysis and/or interpretation of data; implementation of projects and/or experiments
DP	Analysis and/or interpretation of data; implementation of projects and/or experiments
ES	Analysis and/or interpretation of data; statistical analysis; study design
RS	Analysis and/or interpretation of data; final approval of the manuscript; implementation of projects and/or experiments
GPO	Analysis and/or interpretation of data; implementation of projects and/or experiments
RS	Analysis and/or interpretation of data; final approval of the manuscript; implementation of projects and/or experiments
